# Antimicrobial resistance surveillance and trends in armed conflict, fragile, and non-conflict countries of the Eastern Mediterranean Region

**DOI:** 10.1186/s40249-025-01287-8

**Published:** 2025-02-28

**Authors:** Rima Moghnieh, Nazih Bizri, Dania Abdallah, Mohamed H. Sayegh

**Affiliations:** 1 Department of Internal Medicine, Division of Infectious Diseases, Lebanese American University Medical Center-Rizk Hospital, P.O. Box 11-3288, Beirut, Lebanon; 2 Department of Surgery, University of Pittsburgh Medical Center, Pittsburgh, PA 15219 USA; 3 Pharmacy Department, Makassed General Hospital, P.O.Box 11-6301, Beirut, Lebanon; 4 American University of Beirut, P.O. Box 11-0236, Beirut, Lebanon; 5 GAP Solutions (Under Contract No. 75n93019d00026 with National Institute of Allergy and Infectious Diseases, National Institutes of Health, Department of Health and Human Services, United States of America), Washington, USA

**Keywords:** Antimicrobial resistance, Conflict, Fragility, World Health Organization, Eastern Mediterranean Region

## Abstract

**Background:**

The WHO Eastern Mediterranean Region (EMR) faces major social, economic, and demographic challenges, with nearly half of its countries affected by conflicts that severely disrupt health systems. This study compared antimicrobial resistance (AMR) rates and surveillance efforts in conflict-affected, fragile, and non-conflict countries, further subdivided by income.

**Methods:**

Data on bacteriologically confirmed bloodstream infections (BC-BSIs) from 2017 to 2021 were extracted from the WHO GLASS database. Countries were classified as conflict-affected, fragile, or non-conflict (subdivided by income) using World Bank criteria. Descriptive statistics (mean ± SD) were calculated, and group comparisons were performed using unpaired t-tests with Welch’s correction. Mean differences (MD) and 95% confidence intervals (*CI*) were reported.

**Results:**

Conflict-affected countries reported significantly fewer surveillance sites than non-conflict countries (MD: 0.60, 95% *CI:* 0.361 to 0.836, *P* < 0.001) and fewer BC-BSIs per million population (MD: 31.00, 95% *CI:* 17.210 to 44.790, *P* < 0.001). In conflict zones, *Acinetobacter* spp. and *S. aureus* represented a higher proportion of BSIs compared to non-conflict countries (*Acinetobacter* spp. MD: -11.86, 95% *CI:* − 27.130 to 3.399, *P* = 0.099; *S. aureus* MD: − 10.68, 95% *CI:* − 30.030 to 8.680, *P* = 0.203). Carbapenem resistance in *Acinetobacter* spp. exceeded 65% across the groups, peaking in fragile zones (83.38%). Third-generation cephalosporin-resistant *E. coli* (3GCREC) prevalence ranged from 47.99% to 76.34%, peaking in conflict zones (76.34%). Carbapenem-resistant *E. coli* (CREC) prevalence ranged from 2.31% to 15.95%, highest in non-conflict low-middle income countries (15.95%). Third-generation cephalosporin-resistant *K. pneumoniae* (3GCRKP) exceeded 50% in all groups, peaking in conflict zones (80.42%). The prevalence of carbapenem-resistant *K. pneumoniae* (CRKP) ranged from 14.49% to 45.70%, peaking in conflict zones and non-conflict low-middle income countries (45.70%). Methicillin-resistant *S. aureus* (MRSA) exceeded 30%, peaking in conflict zones (70.09%).

**Conclusions:**

Conflict-affected countries have weaker AMR surveillance and lower BC-BSI detection but a higher burden of resistant pathogens, notably carbapenem-resistant *Acinetobacter* spp. and MRSA. Tailored strategies are essential to restore infrastructure, strengthen surveillance, and mitigate the long-term impact of AMR in these zones.

**Graphical Abstract:**

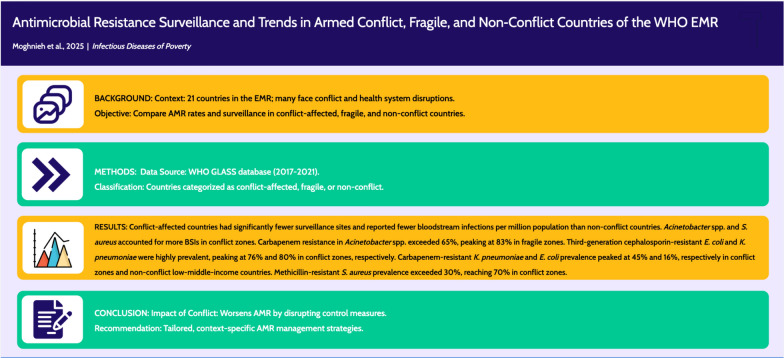

**Supplementary Information:**

The online version contains supplementary material available at 10.1186/s40249-025-01287-8.

## Background

Antimicrobial resistance (AMR) represents a complex and escalating global challenge, often referred to as a “silent pandemic” [1]. It is recognized as one of the top ten public health threats of the twenty-first century [1]. It was estimated that nearly 5 million deaths annually are attributed to drug-resistant bacteria, with the burden disproportionately impacting low- and middle-income countries [2]. A recent study by Talaat et al. examining the rising AMR in the Eastern Mediterranean Region (EMR) of the World Health Organization (WHO) from 2017 to 2019, revealed significant levels of carbapenem-resistant *Acinetobacter* spp. (70.3%) and *Klebsiella pneumoniae* resistant to third-generation cephalosporins (66.3%), thus highlighting a continuous increase that poses a substantial threat to the region’s health security [3].

The WHO EMR has encountered unprecedented challenges, driven by both natural and man-made hazards, severely disrupting health systems and impeding the delivery of adequate healthcare services [4]. This disruption has resulted in increased morbidity and mortality rates. Notably, nine of the region's 22 member states are affected by armed conflict and institutional fragility, contributing to over 40% of global humanitarian needs and more than 60% of the world's refugees, despite the EMR constituting only about 10% of the global population [4–8].

The EMR has a population of approximately 766 million and is characterized by significant cultural, ethnic, and socioeconomic diversity [9, 10]. According to 2021 World Bank data, the region comprises six high-income, 11 middle-income, and five low-income countries. These socioeconomic disparities, coupled with geopolitical tensions and ongoing conflicts, exacerbate public health crises, particularly in lower-income and fragile nations [4].

Armed conflicts, wars, and population displacement further strain already vulnerable healthcare systems, creating conditions conducive to the spread of AMR [3, 4]. This complex interplay of factors highlights the unique challenges faced by the EMR in addressing AMR, particularly in conflict-affected and fragile states.

In response to this growing threat, the WHO and several international scientific organizations have formulated strategic roadmaps to combat AMR and mitigate its progression [3]. Within the EMR, the WHO has actively engaged member states, encouraging the development of five-year national action plans for AMR control [3].

Surveillance is one of the five key pillars identified by the WHO in its global and national action plans to combat AMR [11]. Acknowledging that many countries lacked robust AMR data, the WHO launched the Global Antimicrobial Resistance Surveillance System (GLASS) in 2015 [12]. To assess the regional burden of AMR and generate reliable data, the WHO has supported countries in establishing and enhancing their national AMR surveillance systems [12].

This study aimed to compare AMR rates for bacterial infections caused by priority organisms reported to the WHO through GLASS from 2017 to 2021 across the EMR. We focused on three distinct groups of countries: those affected by conflict, fragile states characterized by institutional and social vulnerabilities, and countries unaffected by conflict or fragility (further categorized as high-income or low/middle-income). We also evaluated AMR surveillance efforts and activities within these groups.

## Methods

### Country classification based on World Bank criteria

Countries were categorized as either “conflict-affected” or “fragile” according to the World Bank’s annual list of “fragile and conflict-affected situations” [13, 14]. Fragile countries were identified based on indicators of weak governance and institutional capacity, which hinder state functions, stability, and development [13, 14]. Conflict-affected countries were defined by a threshold of conflict-related deaths relative to the population, reflecting acute insecurity due to violence by state or non-state actors with political motives, including armed clashes and civilian targeting [13, 14]. Countries that were neither classified as fragile nor conflict-affected were further categorized by income level based on 2021 World Bank data to high-income and to low-middle income (Table 1).

**Table 1:** Country classification based on World Bank criteria

Country Category	Countries included
Fragile (from 2017 to 2021)	Lebanon and Occupied Palestinian Territories (West Bank and Gaza)
Conflict-affected (from 2017 to 2021)	Afghanistan, Iraq, Libya, Somalia, Sudan, Syria, and Yemen
Non-conflict, high-income	Bahrain, Kuwait, Oman, Qatar, Saudi Arabia, and United Arab Emirates
Non-conflict, low- and middle-Income	Djibouti, Egypt, Iran, Jordan, Morocco, Pakistan, and Tunisia

### WHO GLASS database design

Each country designates a variable number of sentinel surveillance sites based on the availability of high-quality microbiology laboratories [3, 12]. AMR data is collected from clinical specimens, such as blood, urine, and stool, which are processed for antimicrobial sensitivity testing to identify bacterial isolates [3, 12]. Reporting follows guidelines from either the Clinical Laboratory Standards Institute (CLSI) or the European Committee on Antimicrobial Sensitivity Testing (EUCAST) [3, 12]. Surveillance sites use WHONET software, which has been modified to streamline data entry and allow for aggregated reporting to GLASS [3, 12].

### AMR surveillance data collection and definitions

In this study, we used publicly available AMR surveillance data extracted from the GLASS global AMR database and country, territory, or area-specific dashboards for WHO EMR countries, covering the period from 2017 to 2021 [3, 15–17].

Key data points included the number of sentinel surveillance sites reporting to GLASS and the total number of bacteriologically confirmed (BC) bloodstream infections (BSIs) (BC-BSIs) reported, serving as surrogate indicators of active AMR surveillance. BSIs were prioritized in the analysis due to their significance as part of the Sustainable Development Goal indicators for AMR. The analysis focused on BC-BSIs caused by specific priority organisms with predefined resistance phenotypes, including *Escherichia coli*, *Klebsiella pneumoniae*, *Acinetobacter* spp., and *Staphylococcus aureus*. The priority resistance phenotypes analyzed included third-generation cephalosporin (3GC)-resistant (*E. coli*, *K. pneumoniae*), carbapenem-resistant (*E. coli*, *K. pneumoniae*, *Acinetobacter* spp.), and methicillin-resistant *Staphylococcus aureus* (MRSA). Population data for each country and year were sourced from the World Bank database.

Data for different outcomes were aggregated for each group of countries (conflict-affected, fragile, non-conflict, non-conflict high-income, and non-conflict low/middle-income) for the period 2017–2021. The endpoints analyzed for each category of countries included:

- *AMR surveillance activities:*
  1. The mean number of sentinel surveillance sites reporting to GLASS per million population for the period 2017–2021.
  2. The mean number of BC-BSIs per million population for the period 2017–2021.
- *Burden of clinically significant bacteria causing BSI*
  1. The percentage of BC-BSIs caused by *Acinetobacter* spp. out of the total number of reported BC-BSIs, calculated as the mean value for the period 2017–2021.
  2. The percentage of BC-BSIs caused by *S. aureus* out of the total number of reported BC-BSIs, calculated as the mean value for the period 2017–2021.
- *Priority organisms resistance phenotypes*
  1. The percentage of BC-BSIs caused by carbapenem-resistant *Acinetobacter* spp*.* (CRAsp) out of the total number of reported BC-BSIs caused by *Acinetobacter* spp*.* with antibiotic susceptibility testing (AST) results (susceptible, intermediate, or resistant) for carbapenems, calculated as the mean value for the period 2017–2021.
  2. The percentage of BC-BSIs caused by 3GC-resistant *E. coli* (3GCEC) out of the total number of reported BSIs caused by *E. coli* with AST results for 3GCs, calculated as the mean value for the period 2017–2021.
  3. The percentage of BC-BSIs caused by carbapenem-resistant *E. coli* (CREC) out of the total number of reported BSIs caused by *E. coli* with AST results for carbapenems, calculated as the mean value for the period 2017–2021.
  4. The percentage of BC-BSIs caused by 3GC-resistant *K. pneumoniae* (3GCRKP) out of the total number of reported BSIs caused by *K. pneumoniae* with AST results for 3GCs, calculated as the mean value for the period 2017–2021.
  5. The percentage of BC-BSIs caused by carbapenem-resistant *K. pneumoniae* (CRKP) out of the total number of reported BSIs caused by *K. pneumoniae* with AST results for carbapenems, calculated as the mean value for the period 2017–2021.
  6. The percentage of BC-BSIs caused by methicillin-resistant *S. aureus* (MRSA) out of the total number of reported BSIs caused by *S. aureus* with AST results for oxacillin/cefoxitin, calculated as the mean value for the period 2017–2021.

It is important to note that for endpoints c)1 to c)6, observations with fewer than 10 BC-BSIs caused by a resistant phenotype per country per year were excluded, in accordance with the WHO GLASS database guidelines.

### Statistical analysis

Data were analyzed to compare the different outcomes across three groups: conflict-affected countries, fragile countries, and countries not affected by conflict or fragility (the latter further subdivided into high-income and low/middle-income subgroups). The normality of data distribution within each group was assessed and confirmed, justifying the use of parametric tests. Descriptive statistics, including mean values and standard deviations (SD), were calculated for each group to evaluate differences in AMR surveillance activity indicators, burden of BC-BSI, and resistance phenotype prevalence. The number of reporting countries for each outcome and country category was summarized using median values and range. Group comparisons were conducted using unpaired *t*-tests, with Welch’s correction applied where necessary to account for unequal variances. Mean differences (MD) between groups were calculated along with 95% confidence intervals (*CI*) to assess the precision of the estimates. A *P*-value of < 0.05 was considered statistically significant. To evaluate the robustness of the findings, sensitivity analyses were conducted using two approaches across all outcomes. First, analyses were restricted to data from 2019 to 2021, excluding earlier years (2017 and 2018), to assess whether trends and group comparisons remained consistent when focusing on more recent data. Second, for the full study period (2017–2021), a random subset of countries was selected from each group to determine whether the results were influenced by specific countries. This involved randomly selecting three countries from the conflict-affected group (out of six), the non-conflict high-income group (out of six), and the conflict middle-income group (out of seven). Due to the limited number of countries in the fragile group (*n* = 2), all available data from this category were retained. These approaches ensured a thorough evaluation of the stability of resistance outcomes and surveillance activities under different scenarios. Microsoft Excel Version 16.77 (Microsoft Corporation, Redmond, WA, USA) was used for data management and preliminary calculations to ensure accuracy. All statistical analyses were conducted using IBM SPSS Statistics for Windows (version 23.0, Armonk, NY, USA: IBM Corp.) and GraphPad Prism version 10.2.1 (GraphPad Software, Inc., San Diego, CA, USA).

## Results

**Fig. 1:**
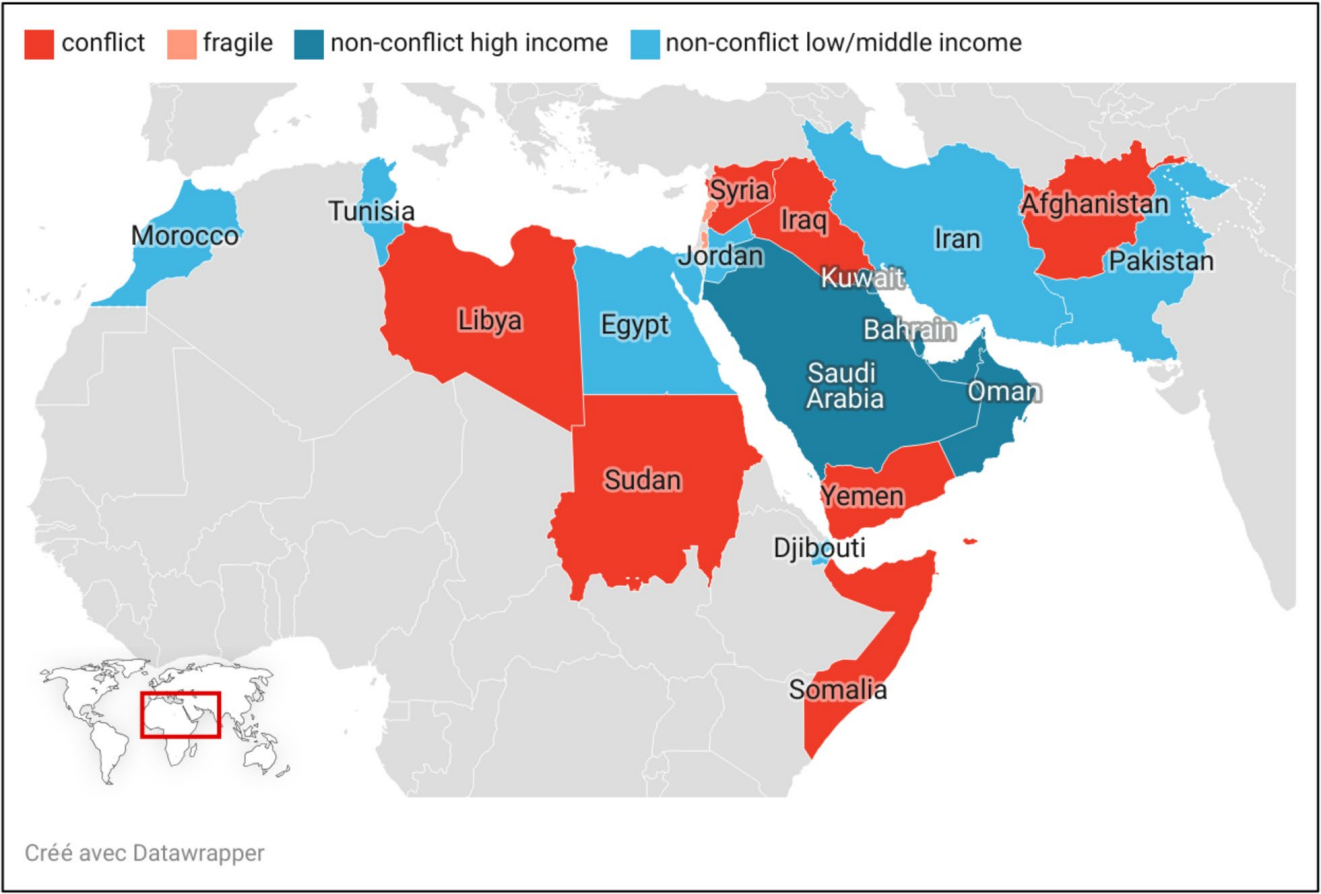
Countries in the state of Conflict and Fragility* in WHO Eastern Mediterranean Region. N.B. Map created by the authors using Datarapper™ (https://www.datawrapper.de/). *The identification as fragile and conflict affected and non-conflict affected is based on the World Bank classification, 2017–2021

From 2017 to 2021, based on the World Bank's classification, 9 out of the 22 countries in the EMR were categorized as either “conflict-affected” (7 countries) or “fragile” (2 countries) (Figure 1). The remaining 13 countries, which were neither fragile nor conflict-affected, were further categorized by income level: high-income (6 countries) and low/middle-income (7 countries).

### AMR surveillance

#### Sentinel surveillance sites reporting to GLASS

**Fig. 2:**
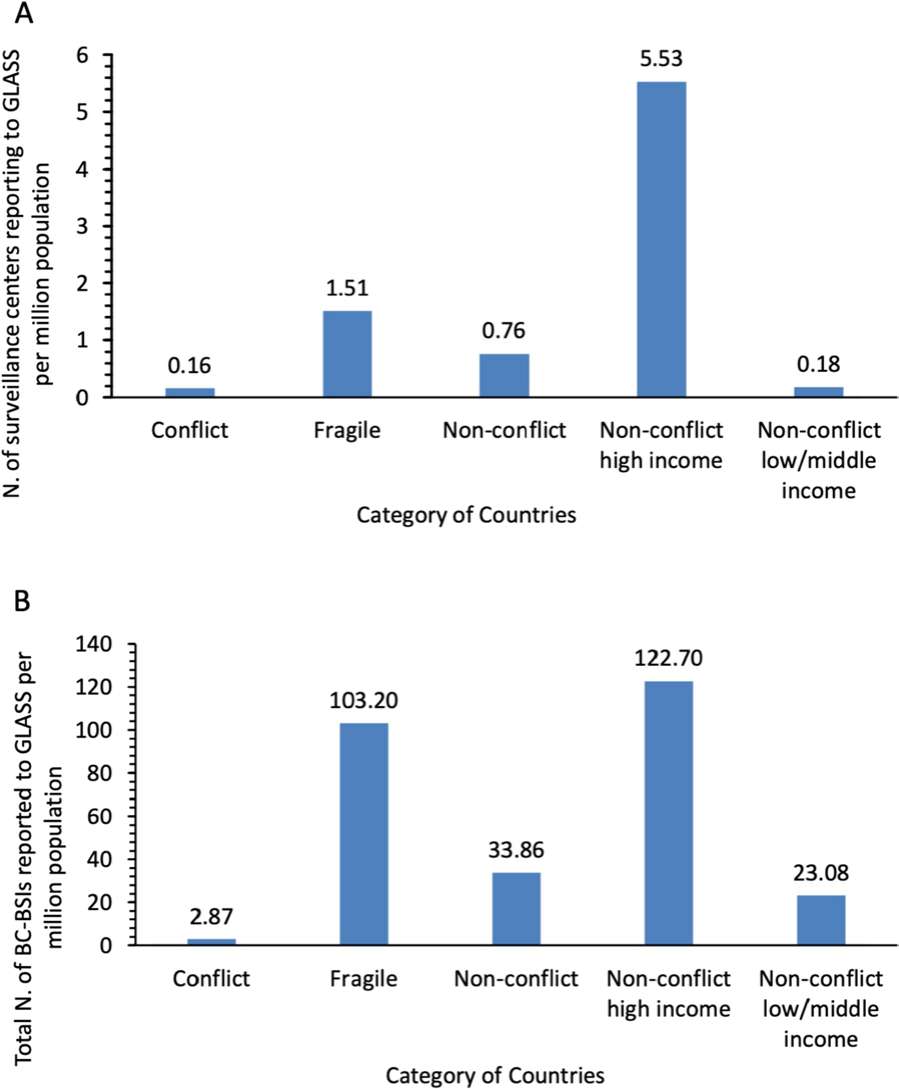
Antimicrobial Resistance (AMR) Surveillance Activities in WHO Eastern Mediterranean Region Countries, Classified by Conflict and Fragility Status**:** **A** Mean Number of Sentinel Surveillance Sites Reporting to GLASS per Million Population (2017–2021); **B** Mean Number of Bacteriologically Confirmed (BC) Bloodstream Infections (BSIs) *(BC-BSIs)* Reported to GLASS per Million Population (2017–2021)

The mean number of sentinel surveillance sites reporting to GLASS per million population for each category is detailed in Supplementary Table S1 and Figure 2A, with group comparisons provided in Supplementary Table S2.

Conflict-affected countries reported significantly fewer surveillance sites than non-conflict countries, (MD: 0.60, 95% *CI:* 0.361 to 0.836, *P* < 0.001). The gap widened further when comparing conflict-affected countries to non-conflict high-income countries (MD: 5.37, 95% *CI:* 3.772 to 6.958, *P* < 0.001). However, the difference between conflict-affected countries and non-conflict low/middle-income countries was minimal (MD: 0.02, 95% *CI:* − 0.111 to 0.151 *P* = 0.736).

Fragile countries generally had more reporting centers compared to conflict-affected nations (MD: − 1.35, 95% *CI:* − 2.422 to − 0.270, *P* = 0.025). However, fragile countries had more centers than non-conflict countries (MD: − 0.75, 95% *CI:* − 1.813 to 0.318, *P* = 0.128). When comparing fragile states to non-conflict high-income countries, the gap was substantial in favor of the later (MD: 4.02, 95% *CI:* 2.420 to 5.619, *P* < 0.001). Interestingly, fragile countries outperformed non-conflict low/middle-income countries (MD: − 1.33, 95% *CI:* − 2.401 to − 0.251, *P* = 0.027).

#### Bacteriologically confirmed BSIs

The mean number of bacteriologically confirmed BSIs reported per million population for each category is presented in Supplementary Table S1 and illustrated in Figure 2B, with group comparisons detailed in Supplementary Table S2.

Conflict-affected countries reported fewer bacteriologically confirmed BSIs per million population than non-conflict countries, (MD: 31.00, 95% *CI:* 17.210 to 44.790, *P* = 0.003). The gap widened further when comparing conflict-affected countries to non-conflict high-income countries (MD: 119.80, 95% *CI:* 86.370 to 153.300, *P* < 0.001).

Fragile countries generally reported more bacteriologically confirmed BSIs per million population compared to conflict-affected nations (MD: − 100.30, 95% *CI:* − 191.500 to − 9.176, *P* = 0.038). When comparing fragile states to non-conflict high-income countries, the difference was in favor of the later (MD: 19.48, 95% *CI:* − 61.420 to 100.200, *P* =0.593). Fragile countries reported more bacteriologically confirmed BSIs per million population than non-conflict low/middle-income countries (MD: − 80.13, 95% *CI:* − 170.900 to 10.630, *P* = 0.071).

### Burden of clinically significant bacteria causing BSI

This study assesses the burden of clinically significant bacteria by examining the percentage of *Acinetobacter* spp. and *S. aureus* among total BSIs. These percentages highlight the prevalence and impact of these pathogens across different settings.

#### *Acinetobacter* spp.

**Fig. 3:**
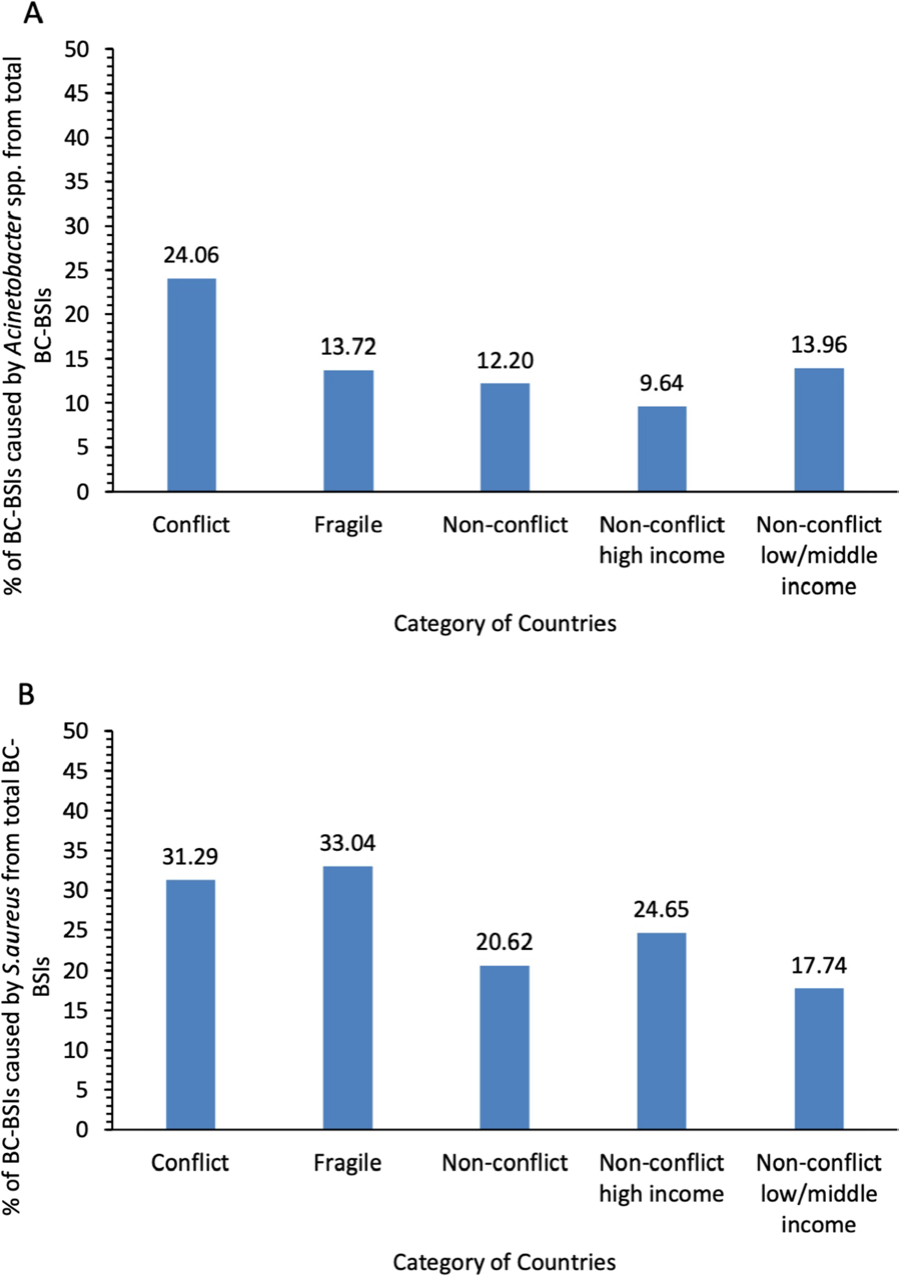
Burden of Clinically Significant Priority Organisms Causing Bacteriologically Confirmed (BC) Bloodstream Infections (BSIs) (BC-BSIs) in WHO Eastern Mediterranean Region Countries, Classified by Conflict and Fragility Status: Mean prevalence (%) of (**A**) *Acinetobacter* spp. and (**B**) *S. aureus* from total BC-BSIs Reported to GLASS (2017–2021)

The mean percentage of *Acinetobacter* spp. among total BSIs for each category is presented in Supplementary Table S1 and Figure 3A, with group comparisons provided in Supplementary Table S2.

Conflict-affected countries reported higher percentages of *Acinetobacter* spp. compared to non-conflict countries (MD: − 11.86, 95% *CI* − 27.130 to 3.399, *P* = 0.099). Fragile countries had a lower percentage of *Acinetobacter* spp. among total BSIs compared to conflict-affected nations (MD: 10.34, 95% *CI* **− **4.010 to 24.690,* P* = 0.135). Compared to non-conflict countries, fragile states showed minimal differences (MD: − 1.52, 95% *CI:* − 8.521 to 5.463, *P* = 0.629).

#### S. aureus

The mean percentage of *S. aureus* among total BSIs for each category is presented in Supplementary Table S1 and Figure 3B, with group comparisons provided in Supplementary Table S2.

Conflict-affected countries reported higher percentages of *S. aureus* compared to non-conflict countries (MD: − 10.68, 95% *CI:* − 30.030 to 8.677, *P* = 0.203). Fragile countries had a slightly higher, yet non-significant, percentage of *S. aureus* compared to conflict-affected countries (MD: − 1.75, 95% *CI:* − 31.990 to 28.490, *P* = 0.897). These countries showed a higher percentage of *S. aureus* compared to non-conflict countries (MD: − 12.43, 95% *CI:* − 43.140 to 18.290, *P* = 0.326).

### Priority organisms resistance phenotypes

The mean prevalence of priority resistance phenotypes, including CRAsp, CREC, 3GCREC, CRKP, 3GCRKP and MRSA, was compared across groups based on conflict status and income level.

#### CRAsp

**Fig. 4:**
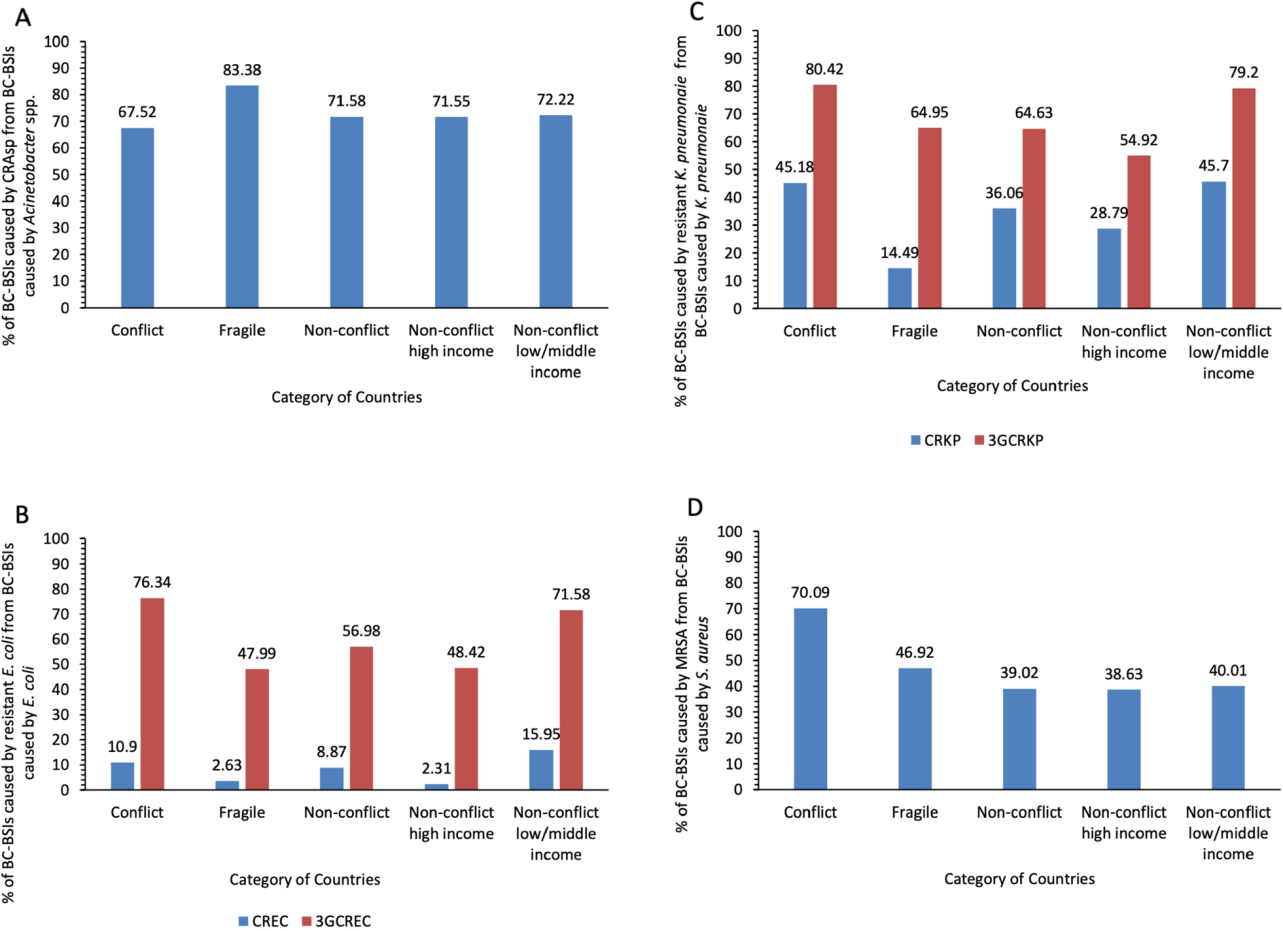
Resistant Priority Organisms Causing Bacteriologically Confirmed (BC) Bloodstream Infections (BSIs) (BC-BSIs) in WHO Eastern Mediterranean Region Countries, Classified by Conflict and Fragility Status (Reported to GLASS, 2017–2021): **A** Mean Prevalence of BC-BSIs Caused by Carbapenem-Resistant *Acinetobacter* spp. (CRAsp) from BC-BSIs Caused by *Acinetobacter* spp.; **B** Mean Prevalence of BC-BSIs Caused by Carbapenem-Resistant *E. coli* (CREC) and Third-Generation Cephalosporin-Resistant *E. coli* (3GCREC) from BC-BSIs Caused by *E. coli*; **C** Mean Prevalence of BC-BSIs Caused by Carbapenem-Resistant *K. pneumoniae* (CRKP) and Third-Generation Cephalosporin-Resistant *K. pneumoniae* (3GCRKP) from BC-BSIs Caused by *K. pneumoniae*; **D** Mean Prevalence of BC-BSIs Caused by Methicillin-Resistant *S. aureus* (MRSA) from BC-BSIs Caused by *S. aureus*

The mean prevalence of CRAsp for each group is detailed in Supplementary Table S1 and Figure 4A, with group comparisons provided in Supplementary Table S2.

In all groups—conflict-affected countries, fragile states, and both high-income and low/middle-income non-conflict settings—the prevalence of CRAsp exceeded 65%, peaking in fragile zones (83.38%). Comparisons reveal that conflict-affected countries had a slightly lower CRAsp prevalence compared to non-conflict areas (MD: 4.06, 95% *CI:* − 14.930 to 23.050, *P* = 0.566).

Fragile countries, however, exhibited a higher prevalence of CRAsp compared to conflict-affected countries (MD: − 15.86, 95% *CI:* − 31.570 to − 0.149, *P* = 0.048). They also had a higher prevalence of CRAsp compared to non-conflict countries (MD: − 11.80, 95% *CI:* − 20.490 to − 3.112, *P* = 0.014).

#### 3GCREC and CREC

The mean prevalence of 3GCREC and CREC across different groups is detailed in Supplementary Table S1 and Figure 4B, with group comparisons provided in Supplementary Table S2.

For 3GCREC, all groups—conflict-affected countries, fragile states, and both high-income and low/middle-income non-conflict settings—reported a prevalence exceeding 45%, ranging from 47.99% to 76.34%, peaking in conflict zones (76.34%). Conflict-affected countries showed a higher prevalence of 3GCREC compared to non-conflict countries (MD: − 19.36, 95% *CI:* − 45.670 to 6.939, *P* = 0.103). Fragile states exhibited a significantly lower 3GCREC prevalence than conflict-affected countries (MD: 28.35, 95% *CI:* 1.850 to 54.860, *P* = 0.042). Fragile states had a significantly lower prevalence of 3GCREC compared to non-conflict countries (MD: 8.99, 95% *CI:* 5.141 to 12.840, *P* < 0.001).

For CREC, in all settings—conflict-affected countries, fragile states, and both high-income and low/middle-income non-conflict areas—the prevalence ranged from 2.31% to 15.95%, highest in non-conflict low-middle income countries (15.95%). When comparing conflict-affected countries with non-conflict countries, the prevalence of CREC was higher in conflict settings (MD: − 2.04, 95% *CI:* − 11.070 to 7.005, *P* = 0.618), yet lower than non-conflict low/middle-income countries (MD: 5.05, 95% *CI:* − 4.520 to 14.620, *P* = 0.258). Fragile states exhibited a lower CREC prevalence than conflict-affected countries (MD: 7.27, 95% *CI:* − 2.829 to 17.360, *P* = 0.118), and non-conflict countries overall (MD: 5.23, 95% *CI:* 1.694 to 8.770, *P* = 0.009).

#### CRKP and 3GCRKP

The mean prevalence of 3GCRKP and CRKP across different groups is detailed in Supplementary Table S1 and Figure 4C, with group comparisons provided in Supplementary Table S2.

For 3GCRKP, all groups—conflict-affected countries, fragile states, and both high-income and low/middle-income non-conflict settings—reported a prevalence exceeding 50%, ranging from 54.92% to 80.42%, peaking in conflict zones (80.42%). Conflict-affected countries showed a higher prevalence of 3GCRKP compared to non-conflict countries (MD: − 15.79, 95% *CI:* − 25.99 to − 5.59, *P* = 0.0091). Fragile states showed a significantly lower prevalence of 3GCRKP than conflict-affected countries (MD: 15.47, 95% *CI:* 1.709 to 29.230, *P* = 0.033). Fragile states exhibited a higher prevalence of 3GCRKP compared to non-conflict high-income countries (MD: − 10.04, 95% *CI:* − 21.790 to 1.720, *P* = 0.085). In contrast, fragile states demonstrated a significantly lower prevalence of 3GCRKP compared to non-conflict low/middle-income countries (MD: 14.25, 95% *CI:* 3.390 to 25.110, *P* = 0.021).

For CRKP, in all settings—conflict-affected countries, fragile states, and both high-income and low/middle-income non-conflict areas—the prevalence of ranged from 14.49% to 45.70%, peaking in conflict zones and non-conflict low-middle income countries (45.70%). Conflict-affected countries had a higher prevalence of CRKP compared to non-conflict countries (MD: − 9.12, 95% *CI:* − 75.990 to 57.750, *P* = 0.635). Fragile states exhibited a lower prevalence of CRKP compared to conflict-affected countries (MD: 30.69, 95%* CI:* − 36.400 to 97.780, *P* = 0.197). Comparing fragile states to non-conflict groups revealed a significantly lower prevalence of CRKP in the former (MD: 21.57, 95% *CI:* 11.350 to 31.790, *P* = 0.001).

#### MRSA

The mean prevalence of methicillin-resistant *Staphylococcus aureus* (MRSA) across different groups is presented in Supplementary Table S1 and Figure 4D, with group comparisons provided in Supplementary Table S2.

In all settings—conflict-affected countries, fragile states, and both high-income and low/middle-income non-conflict areas—the prevalence of MRSA exceeded 30%, ranging from 38.63% to 70.09%. Conflict-affected countries exhibited a significantly higher prevalence of MRSA compared to non-conflict countries, with an MD of − 31.07% (95% *CI:* − 70.880 to 8.746, *P* = 0.081). Fragile states had a lower prevalence of MRSA compared to conflict-affected countries (MD: 23.17, 95% *CI:* − 28.910 to 75.250, *P* = 0.318). When comparing fragile countries to non-conflict countries, the prevalence of MRSA was higher in the former (MD: − 7.90, 95% *CI:* − 49.340 to 33.540, *P* = 0.629), regardless of income level.

## Sensitivity analysis of different outcomes

For the AMR surveillance activities and the burden of clinically significant bacteria causing BC-BSI, sensitivity analyses confirmed the stability of findings across most group comparisons (supplementary Tables S3–6). There was no variability in results when using the random country selection approach or focusing on different time periods. For example, sensitivity analyses confirmed the robustness of the main findings regarding sentinel surveillance sites reporting to GLASS across both methods of data analysis. Using the random country selection approach (2017–2021), conflict-affected countries continued to report significantly fewer sentinel surveillance sites per million population compared to non-conflict countries (MD: 0.84, 95% *CI:* 0.564 to 1.108, *P* < 0.001). When comparing fragile countries to conflict-affected countries, the results also supported the main analysis, indicating that fragile countries had more surveillance sites (MD: − 1.31, 95% *CI:* − 2.383 to − 0.233, *P* = 0.028). These trends were further reinforced by the second sensitivity analysis focusing on a more restricted time period (2019–2021), where the differences in surveillance site reporting were consistent across all country categories. For the percentage resistance outcomes, data was restricted to the years 2019 to 2021 due to inconsistent data availability from conflict-affected countries (*n* = 6), where typically only 1–2 countries reported each data point annually, making it challenging to perform meaningful analyses across the full time period. Despite this limitation, sensitivity analysis using data from 2019–2021 yielded results that were consistent with the main analysis (2017–2021), reinforcing the robustness of the observed trends (supplementary Tables S5 and S6). It is important to note that for all comparisons across all outcomes, due to the limited number of countries in the fragile group (*n* = 2), all available data from this category were retained. Refer to supplementary Tables S3-S6 for other group comparisons and outcomes.

## Discussion

### An overview of AMR surveillance challenges in conflict-affected zones

This study revealed significant disparities in AMR surveillance across different country categories—conflict-affected, fragile, and non-conflict—highlighting the impact of income levels and armed conflict on surveillance capacity. Conflict-affected countries report the fewest number of sentinel surveillance centers reporting to GLASS and the lowest incidence of BC-BSIs per million population. This reflects the profound disruptions to healthcare infrastructure in war-torn regions. Fragile states, though not directly involved in active conflict, face similar challenges due to political instability and weak health systems. In contrast, non-conflict countries, particularly high-income nations, have more centers and report more BC-BSIs. However, income levels remain a critical determinant of surveillance performance.

AMR surveillance is a complex task requiring trained microbiologists, well-equipped laboratories, and a robust system for collecting, compiling, and managing data from multiple laboratories, which is then fed into specialized databases like GLASS. However, armed conflicts deplete resources and often dismantle these systems. Syria serves as a prominent example, where the destruction of health facilities, the exodus or death of healthcare workers, and the fragmentation of the health system have significantly hindered AMR surveillance efforts [18].

Reliable data on AMR in conflict zones like Syria are scarce, with available studies limited by small sample sizes and a lack of generalizability [18, 19]. For instance, a study by Karamya et al. analyzing bacteriology data from public and private hospitals in major Syrian cities reported high rates of antibiotic resistance, particularly among Gram-negative bacteria, despite inconsistent testing and a lack of standardized procedures [19]. *Acinetobacter* spp. demonstrated notable resistance, with 90% of isolates resistant to meropenem and 85% to amikacin [19]. The study also revealed inaccuracies, such as testing antibiotics against bacteria with intrinsic resistance or where no CLSI breakpoints existed, leading to erroneous results, especially for *Pseudomonas aeruginosa* and tigecycline [19]. Such inconsistencies in antimicrobial susceptibility testing (AST), combined with the absence of computerized data systems, complicate surveillance and increase the risk of errors [19]. These findings point to alarmingly high levels of AMR in Syria, worsened by inconsistencies in laboratory practices, and emphasize on the urgent need for improved AST methods, laboratory infrastructure, and data management in conflict-affected regions.

### The link between inadequate infection prevention and control (IPC) and water, sanitation, and hygiene (WASH) services and the spread of AMR in conflict zones

In this study, the prevalence of *Acinetobacter* spp. in BSIs was highest in conflict-affected countries, a trend linked to compromised infection prevention and control (IPC) measures in these regions. *Acinetobacter* spp., a pathogen notorious for its environmental persistence, requires stringent IPC measures, including effective environmental cleaning, to control its spread [20, 21]. Armed conflict significantly impairs IPC efforts, which are essential for preventing infections in healthcare settings. Effective IPC relies on leadership support, healthcare worker education, performance monitoring, and feedback, all of which become challenging to implement in conflict-affected and fragile areas [22]. In a previous study, we evaluated IPC education and training across WHO EMR member states, revealing fragmented and inconsistent practices, particularly in conflict-affected and fragile countries [23]. These nations often prioritize IPC education less during crises, with reduced training and the hiring of less experienced staff compromising infection control efforts [23]. Similar challenges were observed in conflict-affected areas in northeastern Syria, where a study involving 33 healthcare facilities revealed severe deficiencies in IPC compliance, with above 80% of these many facilities failing to meet WHO standards [24]. Such gaps in IPC programs and training exacerbate healthcare-associated infections and contribute to the spread of resistant pathogens.

In this study, conflict-affected areas exhibited higher rates of antibiotic resistance compared to non-conflict regions, particularly in CRAsp and MRSA. This trend aligned with findings from a systematic review by Truppa et al., who reported elevated levels of carbapenem resistance in *Acinetobacter* spp. (74%), significant resistance in Enterobacteriaceae, and a median methicillin resistance of 45% in *S. aureus* across the Middle East [25].

The spread of AMR is significantly exacerbated by inadequate water, sanitation, and hygiene (WASH) services, especially in conflict zones where infrastructure is often destroyed. In community settings, IPC depends on vaccination programs, robust health and sanitation infrastructure, and proper water and waste management [26]. In healthcare facilities, adherence to IPC standards—such as hand hygiene, environmental cleaning, and isolating patients with resistant organisms—is crucial to preventing the spread of AMR [27, 28]. However, in high-conflict areas, health and sanitation infrastructure often suffer extensive damage, disrupting medical supplies and undermining regulatory policies critical for infection control. In such areas with limited resources, the absence of WASH services leads to poor sanitation conditions, restricted access to clean water, and insufficient hygiene practices, all of which contribute to the spread of resistant bacteria and the escalation of AMR [29]. For example, the destruction of water and sewage systems in conflict zones like Gaza severely compromises water quality and sanitation [30]. Shrapnel from explosives can damage water treatment facilities, leading to interrupted water supplies and compromised sterile conditions in hospitals [30]. This, in turn, increases post-surgical infections and facilitates the spread of communicable diseases, creating conditions conducive to the development of drug-resistant pathogens [30]. A 2017 study in Iraq revealed significant antibiotic contamination in potable water, with ciprofloxacin, levofloxacin, and amoxicillin detected in both untreated and treated water from Baghdad’s treatment plants. This raises serious concerns about the potential contribution to antibiotic resistance [31]. Similarly, a 2021 pilot study in Gaza identified high levels of antimicrobial-resistant bacteria, including ESBL and carbapenemase-producing strains, in water, wastewater, and surface samples from hospitals. This highlights the critical need for effective WASH services in healthcare settings to combat AMR [32]. Overall, the direct and indirect impacts of conflict on urban public services, such as water and sewage systems, significantly affect AMR. The destruction and disruption of these essential services compromise hygiene and infection control, fostering environments where resistant pathogens can thrive and threatening community health for years [33]. Addressing these issues requires a concerted effort to restore and maintain infrastructure and improve IPC practices to effectively mitigate the spread of AMR.

### The overuse and misuse of antibiotics in conflict zones and the role of antibiotic stewardship

The use of antibiotics is another key driver of AMR, particularly in conflict zones where access to appropriate antibiotic formulations is limited, and regulation is often poor. Studies from the Palestinian territories and Gaza have highlighted excessive antibiotic use in healthcare settings, contributing to rising resistance levels [34, 35]. In a study examining 428 inpatient prescriptions from two major government hospitals, findings revealed an average of 6.72 ± 4.37 drugs per prescription, with 40% being antibiotics [34]. This high level of antibiotic consumption exceeds optimal standards, indicating overuse and a potential risk for AMR development [34]. Similarly, a study from hospitals in the Gaza Strip found inconsistent availability of key antibiotics, with frequent stockouts linked to the ongoing blockade and poor adherence to standard treatment guidelines [35]. This reflects broader regional issues, such as inadequate regulatory enforcement and poor compliance with antibiotic stewardship programs. In many EMR countries, the over-the-counter availability of antibiotics and widespread self-medication complicate efforts to control AMR, as prescription regulation in community settings is weak or nonexistent [36]. For instance, in Iraq, nearly half of community pharmacists dispense antibiotics without a prescription, and self-medication is common [37]. Moreover, conflict zones often experience an influx of poor-quality generic antibiotics [38]. These practices highlight the urgent need for improved antibiotic management and stewardship programs.

In conflict-affected regions with fragmented healthcare systems, enforcing antimicrobial stewardship (AMS) requires tailored, multifaceted approaches to address the unique challenges posed by these environments. Developing local antibiotic treatment guidelines based on local disease and AMR epidemiology is essential to ensure appropriate antibiotic use [39]. Training programs targeting primary care workers, first responders, and specialists should be implemented alongside online AMS education platforms and electronic clinical decision support systems (eCDSS), such as Médecins Sans Frontières (MSF) e-care [39]. Regular monitoring of antibiotic consumption through systematic audits, point-prevalence surveys, and consumption tracking systems is also crucial to identify and address inappropriate prescribing patterns [40]. Empowering pharmacists through AMS training and engaging opinion leaders to promote awareness among healthcare professionals and the public are key strategies to improve stewardship efforts. Successful examples, such as the International Committee of the Red Cross (ICRC) AMS protocol implemented in a reconstructive surgical program in Lebanon, highlight the feasibility of integrating AMS measures, including IPC, prescribing optimization, and laboratory support, even in resource-limited, conflict-affected settings [41]. Collaborative regional efforts, leveraging the expertise and guidance of more advanced AMS programs in countries like Saudi Arabia [42], could further support less-resourced nations in the region. In addition, the establishment of regional antibiotic guidelines informed by shared resistance data would standardize practices and strengthen collective AMR mitigation.

Raising awareness among decision-makers is essential to strengthen legislation on antibiotic use and regulate over-the-counter antibiotic sales, a persistent challenge in most conflict and fragile regions where weak regulatory systems permit unrestricted access [43]. Advocacy efforts should include comprehensive campaigns targeting consumers and healthcare providers, using mass media, social media platforms, and public engagement initiatives to promote AMR awareness across all levels of society [43]. These campaigns should incorporate school-based education, professional training, and digital outreach while empowering healthcare professionals to advocate for rational antibiotic use and contribute to legislative reforms [43]. By combining these targeted interventions, AMS programs can adapt to the complexities of conflict-affected settings, supporting sustainable AMR control efforts in these vulnerable regions.

### The impact of heavy metal contamination on AMR in conflict zones

Exposure to heavy metals from bombings during conflicts has also been linked to the induction of antibiotic resistance [38, 44, 45]. Research suggests that pathogens like *A. baumannii*, often associated with wound infections in conflict zones, may develop resistance in environments contaminated with toxic heavy metals [38, 44, 45]. Field hospitals in Syria, Libya, Yemen, and Iraq have reported numerous cases of wound contamination with resistant bacteria caused by conventional weapons such as bombs, landmines, and missiles, complicating treatment and contributing to the spread of AMR [18, 25, 38, 44–46].

### Addressing AMR in conflict and fragile zones: a context-specific approach with national action plans for AMR as a strategic framework

In this study, fragile countries (2 countries) displayed mixed performance in both AMR surveillance and BC-BSI per million reporting, positioning them between conflict-affected and non-conflict nations. They generally have more sentinel surveillance sites reporting to GLASS compared to conflict-affected countries, indicating better infrastructure, but fewer than non-conflict nations, especially high-income countries. Similarly, fragile states report more BC-BSIs per million and denominator is smaller than conflict-affected countries, suggesting relatively better healthcare access or reporting systems, although they still trail behind high-income nations. Interestingly, fragile countries outperform non-conflict low- and middle-income countries in both surveillance and BC-BSI per million reporting, demonstrating that fragility does not always equate to weaker systems. Factors such as healthcare investments, international aid, and external support may contribute to these differences [10, 14]. These nations report higher prevalence rates of *Acinetobacter* spp. compared to non-conflict low/middle-income countries but lower resistance levels in other pathogens such as *E. coli* and *K. pneumoniae*. Fragile states, characterized by weak institutions and large-scale refugee movements, often experience the introduction of resistant strains from conflict-affected areas, further complicating AMR control efforts [10, 14, 47–49]. Despite the relative strengths, fragile nations still face significant challenges due to resource limitations and political instability, which affect their ability to fully curb AMR. In contrast, non-conflict countries, particularly high-income nations, exhibit better AMR surveillance and lower resistance rates compared to their low/middle-income counterparts. High-income countries benefit from superior healthcare infrastructure and consistent reporting, resulting in lower resistance rates in key pathogens. However, addressing AMR in low/middle-income countries will require significant investments in infrastructure and strong political commitment to address the underlying factors that drive resistance [49].

Addressing AMR in fragile and conflict zones in the Middle East requires context-specific strategies that account for the challenges posed by conflict and fragility. These strategies should focus on restoring infrastructure and strengthening IPC programs to mitigate the spread of resistant pathogens.

As previously mentioned, conflict and fragile countries in the EMR are heterogeneous, differing significantly in terms of political, economic, and health system structures, as well as the extent to which they have been affected by conflict. Addressing AMR in these diverse situations, although it follows general guidelines, requires tailoring interventions to each country’s unique circumstances. The broader strategies are already outlined in the National Action Plans (NAPs) developed with support from the WHO Eastern Mediterranean Regional Office.

The NAP on AMR typically follows the framework set by the WHO, which is built around five key pillars [50]. These pillars provide a comprehensive strategy for tackling AMR at the national level:

1. Improving awareness and understanding of AMR
2. Developing and strengthening AMR surveillance
3. Reducing the incidence of infections through strengthening IPC measures in healthcare and community settings
4. Optimizing the use of antimicrobials
5. Sustainable investment and governance

Each country’s NAP is tailored to its specific context, but these pillars offer a universal roadmap to effectively address AMR. The starting point for implementing each pillar varies between countries and even within the same country, depending on the political, social, and health-related conditions at different points in time.

In conflict-affected areas, AMR surveillance becomes an “unaffordable luxury” due to challenges such as disrupted communication, interrupted internet access, and the lack of physical connectivity between laboratories and hospitals [51]. In addition, these areas face shortages in diagnostic materials and expertise. Sentinel surveillance in specialized clinics established by nongovernmental organizations (NGOs) in or near conflict zones can and have served as indicators of the pattern and extent of circulating AMR in these areas [41, 52–54]. Moreover, developed countries, or non-conflict nations in the region, can support AMR surveillance in similar ways to how the US Center of Disease Control and Prevention has assisted Ukraine [55]. This includes laboratory training, modern lab equipment, standardized testing procedures, and improvement plans, leading to significant advancements in AMR detection [55].

In areas experiencing heavy conflict, providing WASH services becomes a high priority, especially where water supply and sewage disposal systems have been damaged or compromised. Access to clean water, good hygiene practices, and adequate nutrition are critical components of infection prevention. Antiseptic and disinfectant products also become essential targets in these contexts. Governments, often preoccupied with political and military turmoil, rely heavily on NGOs for AMR intervention. Médecins Sans Frontières has developed a context-adapted approach that focuses on high-impact IPC interventions, such as hand hygiene and mobile applications with stepwise IPC improvement tools, which have been effective in facilitating IPC implementation [52]. Hospital bed arrangements that allow cohorting patients with similar infecting organisms and AMR profiles further enhance IPC efforts.

Antimicrobial use in high-conflict areas can become erratic due to several factors: the exodus of medical experts, reliance on what antibiotics are available, and the lack of trauma-specific guidelines. A key intervention in these situations is to provide hospitals and pharmacies with essential antibiotics, especially those in the “Access” category according to the AWARE classification [56]. Increasing local healthcare workers’ awareness about recommended antimicrobial therapy and its duration in various indications can help guide proper use [57]. In addition, vaccination campaigns and providing adequate nutrition play a crucial role in antimicrobial stewardship by preventing childhood infections and avoiding malnutrition, thus reducing unnecessary antibiotic use.

In countries experiencing low-intensity conflict, intermittent conflict, or fragility, political, military, and social challenges often relegate AMR to a lower priority in government agendas. This impacts the budget allocated for AMR activities. These countries differ significantly in their implementation of NAPs—some have yet to begin, while others, like Lebanon, have started updating their AMR NAP for the next five years. Interventions in these countries vary depending on the level of progress made. Prioritizing IPC in the NAP on AMR is essential, including key performance indicators such as hand hygiene compliance, environmental cleaning, equipment sterilization, and aseptic techniques, which should be integrated into hospital and long-term care facility accreditation standards. Special attention must also be given to IPC in the community especially in refugee camps and displaced populations, such as vaccination against preventable diseases like measles, cholera, hepatitis, and other communicable infections, providing adequate and clean water supplies and proper waste disposal [58, 59]. In fragile countries, AMR surveillance can be improved through capacity-building projects, such as training healthcare personnel or implementing point-of-care testing [60].

### Limitations

This study is based on data submitted to the GLASS by member states of the EMR, which may be influenced by variability in data availability, laboratory controls, and hospital types, potentially introducing bias, as previously discussed. The analysis is confined to the EMR, where conflict-affected, fragile, and non-conflict countries are compared. While there might be a temptation to generalize our conclusions to other conflict regions globally, such generalization requires further validation from other conflict-affected areas. For example, data from Ukraine indicate a rise in the emergence and spread of carbapenem-resistant bacteria during conflict times, accompanied by a sharp increase in antibiotic consumption in recent years of high-intensity conflict [61, 62]. However, it's crucial to recognize the unique conditions of each region that could influence these results, making it prudent to examine conflict-affected regions with diverse geopolitical landscapes. The EMR is unique in its geopolitical and economic composition, encompassing countries across a wide economic spectrum, with varying types of regimes and political structures. Fragile and conflict-affected countries within this region have endured a weakened healthcare infrastructure for decades, not just for one or two years, which complicates the generalizability of findings to other global regions. These differences contribute to discrepancies between conflict-affected and non-conflict countries and may inflate the perceived impact of conflict. Another limitation is the selective analysis of pathogens; for example, *Pseudomonas aeruginosa* is not included in the WHO GLASS database, thus not providing a comprehensive picture of AMR. Regarding the sensitivity analysis, the random country selection approach could not be applied for fragile countries (only two countries were available), necessitating a modified time-point approach across all outcomes. For the percentage resistance outcomes in conflict-affected countries (*n* = 6), the random selection approach was not feasible due to inconsistent data availability, leading to the adoption of a modified time-point approach focusing on data from 2019 to 2021. Despite these limitations, the findings provide significant insights into the impact of conflict and fragility on AMR surveillance and prevalence, reflecting the enduring effects of such conditions, as Plato once said, “Only the dead see the end of war.”

## Conclusions

This study highlighted the challenges faced by conflict-affected and fragile countries in AMR surveillance and reporting. These zones report lower rates of BC-BSI per million population and higher prevalence of resistant bacteria compared to non-conflict, high-income countries. The surveillance gap is particularly pronounced in conflict zones, where armed conflicts disrupt essential systems such as IPC, sanitation, and antibiotic stewardship. AMR, therefore, emerges as a significant collateral damage of conflict, with effects that persist long after the violence ends. Addressing AMR in these regions will require context-specific strategies that account for the challenges posed by conflict and fragility, with a focus on restoring infrastructure and strengthening IPC and surveillance systems to mitigate the spread of resistant pathogens.

## Supplementary Information

Supplementary materials 1

## Data Availability

The data that support the findings of this study are publicly available at The World Health Organization Global Antimicrobial Resistance Surveillance System Dashboard.

## Abbreviations

3GC: Third-generation cephalosporin
3GCREC: Third-generation cephalosporin-resistant *E. coli*
3GCRKP: Third-generation cephalosporin-resistant *K. pneumoniae*
AMR: Antimicrobial resistance
AMS: Antimicrobial stewardship
AST: Antibiotic susceptibility testing
BC-BSIs: Bacteriologically confirmed bloodstream infections
*CI*: Confidence interval
CLSI: Clinical Laboratory Standards Institute
CRAsp: Carbapenem-resistant *Acinetobacter* spp.
CREC: Carbapenem-resistant *E. coli*
CRKP: Carbapenem-resistant *K. pneumoniae*
EMR: Eastern Mediterranean Region
EUCAST: European Committee on Antimicrobial Sensitivity Testing
GLASS: Global Antimicrobial Resistance and Use Surveillance System
IPC: Infection prevention and control
Max: Maximum
MD: Mean of differences
Min: Minimum
MNRC: Median Number of Reporting Countries
MRSA: Methicillin-resistant *S. aureus*
N: Number
SD: Standard deviation
WHO: World Health Organization
