# Supplementary material for: Antimicrobial resistance surveillance and trends in armed conflict, fragile, and non-conflict countries of the Eastern Mediterranean Region
Source: Infect Dis Poverty. 2025 Feb 28;14:14. doi: 10.1186/s40249-025-01287-8 (PMC11869426; doi:10.1186/s40249-025-01287-8)
Supplement: Supplementary file 1 — Supplementary materials 1 [file 40249_2025_1287_MOESM1_ESM.docx]

**Supplementary Material**

**Tables**

**Table S1.** Antimicrobial Resistance (AMR) Surveillance Activities, Burden of Clinically Significant Bacteria Causing Bacteriologically Confirmed (BC) Bloodstream Infections (BSIs) (BC-BSIs), and Resistant Priority Organisms Causing BC-BSIs in WHO Eastern Mediterranean Region Countries, Classified by Conflict and Fragility Status (Reported to GLASS, 2017–2021)

| **Outcome** | | | **Category of Countries** | | | | |
| --- | --- | --- | --- | --- | --- | --- | --- |
|  |  |  | **Conflict  (*N* = 7  countries)** | **Fragile (*N* = 2  countries)** | **Non-conflict  (*N* = 13 countries)** | **Non-conflict  high income (*N* = 6 countries)** | **Non-conflict  low/middle income  (*N* = 7 countries)** |
| **AMR Surveillance Activities** | *N. of Sentinel Surveillance Sites per Million Population* | Mean (SD) | 0.16 (0.08) | 1.51(0.87) | 0.76 (0.21) | 5.53 (1.29) | 0.18 (0.10) |
|  |  | MNRC (Min–Max) | 5 (1–7) | 2 (1–2) | 10 (9–12) | 5 (4–6) | 5 (5–6) |
|  | *N. of* BC-BSIs *Reported to GLASS per Million Population* | Mean (SD) | 2.87 (2.41) | 103.2 (73.45) | 33.86 (11.22) | 122.70 (27.00) | 23.08 (9.97) |
|  |  | MNRC (Min–Max) | 5 (1-7) | 2 (1-2) | 11 (9-13) | 5 (4-6) | 6 (5-7) |
| **Burden of Clinically Significant Bacteria Causing BC-BSIs** | Prevalence of Acinetobacter spp. from total BC-BSIs (%) | Mean (SD) | 24.06 (12.41) | 13.72 (6.29) | 12.2 (2.55) | 9.64 (1.17) | 13.96 (3.23) |
|  |  | MNRC (Min–Max) | 3 (1–5) | 1 (1–2) | 10 (7–12) | 5 (3–6) | 5 (4–6) |
|  | Prevalence of S. aureus from total BC-BSIs (%) | Mean (SD) | 31.29 (15.67) | 33.04 (24.79) | 20.62 (2.20) | 24.65 (2.04) | 17.74 (3.97) |
|  |  | MNRC (Min–Max) | 4 (1–6) | 2 (1–2) | 10 (7–12) | 5 (3–6) | 5 (4–6) |
| **Resistant Priority Organisms Causing BC-BSIs** | Prevalence of BC-BSIs  caused by CRAsp  from BC-BSIs  caused by *Acinetobacter* *spp.* (%) | Mean (SD) | 67.52 (12.38) | 83.38 (7.53) | 71.58 (3.77) | 71.55 (5.33) | 72.22 (4.90) |
|  |  | MNRC (Min–Max) | 1 (0–2) | 1 (1–2) | 10 (6–11) | 5 (3–6) | 5 (3–5) |
|  | Prevalence of BC-BSIs  caused by CREC  from BC-BSIs  caused by *E. coli* (%) | Mean (SD) | 10.90 (8.19) | 3.63 (1.43) | 8.87 (3.12) | 2.31 (0.25) | 15.95 (4.36) |
|  |  | MNRC (Min–Max) | 1 (1-2) | 2 (1-2) | 10 (5-11) | 5 (3-6) | 5 (2-5) |
|  | Prevalence of BC-BSIs  caused by 3GCREC  from BC-BSIs  caused by *E. coli* (%) | Mean (SD) | 76.34 (16.79) | 47.99 (2.13) | 56.98 (3.06) | 48.42 (2.98) | 71.58 (7.54) |
|  |  | MNRC (Min–Max) | 1 (0–1) | 2 (1–2) | 10 (6–11) | 5 (3–6) | 5 (3–5) |
|  | Prevalence of BC-BSIs  caused by CRKP  from BC-BSIs  caused by *K. pneumoniae* (%) | Mean (SD) | 45.18 (28.27) | 14.49 (6.87) | 36.06 (7.14) | 28.79 (7.27) | 45.70 (5.62) |
|  |  | MNRC (Min–Max) | 1 (0-2) | 2 (1-2) | 10 (8-11) | 5 (4-6) | 5 (4-5) |
|  | Prevalence of BC-BSIs  caused by 3GCRKP  from BC-BSIs  caused by *K. pneumoniae* (%) | Mean (SD) | 80.42 (4.72) | 64.95 (8.82) | 64.63 (6.15) | 54.92 (7.22) | 79.20 (1.64) |
|  |  | MNRC (Min–Max) | 1 (0-2) | 2 (1-2) | 10 (8-11) | 5 (4-6) | 5 (4-5) |
|  | Prevalence of BC-BSIs  caused by MRSA  from BC-BSIs  caused by *S. aureus* (%) | Mean | 70.09 (17.13) | 46.92 (33.57) | 39.02 (5.14) | 38.63 (3.86) | 40.01(6.76) |
|  |  | MNRC (Min–Max) | 1 (0–2) | 2 (1–2) | 10 (5–11) | 5 (3–6) | 5 (2–6) |

**Abbreviations**: AMR = Antimicrobial Resistance, BC-BSIs = Bacteriologically Confirmed Bloodstream Infections, CRAsp = Carbapenem-Resistant *Acinetobacter* spp., CREC = Carbapenem-Resistant *E. coli*, CRKP = Carbapenem-Resistant *K. pneumoniae*, Max = Maximum, Min = Minimum, MNRC = Median Number of Reporting Countries, MRSA = Methicillin-Resistant *S. aureus*, N = Number, SD = Standard Deviation, 3GCREC = Third-Generation Cephalosporin-Resistant *E. coli*, 3GCRKP = Third-Generation Cephalosporin-Resistant *K. pneumoniae*.

**N.B.**

- For each outcome and category of countries, mean values and SD were calculated.
- The number of reporting countries for each outcome and category was summarized using median values and ranges.

**Table S2.** Group Comparisons of Countries Classified by Conflict and Fragility Status Across Study Outcomes Related to Antimicrobial Resistance Surveillance Activities, Burden of Clinically Significant Bacteria Causing Bacteriologically Confirmed Bloodstream Infections, and Resistant Priority Organisms in WHO Eastern Mediterranean Region Countries (Reported to GLASS, 2017–2021)

| **Outcome** | | | **Group Comparisons between categories of countries (Group A vs. B) (A-B)*** | | | | | | | |
| --- | --- | --- | --- | --- | --- | --- | --- | --- | --- | --- |
|  |  |  | **Non-conflict**  **vs.**  **Conflict** | **Non-conflict  high income**  **vs.**  **Conflict** | **Non-conflict  low/**  **middle income**  **vs.**  **Conflict** | **Non-conflict**  **vs.**  **Fragile** | **Non-conflict  high income**  **vs.**  **Fragile** | **Non-conflict  low/**  **middle income**  **vs.**  **Fragile** | **Conflict**  **vs.**  **Fragile** | **Non-conflict  high income**  **vs.**  **Non-conflict  low/**  **middle income** |
| **AMR Surveillance Activities** | *N. of Sentinel Surveillance Sites per Million Population* | MD ± SEM | 0.60 ± 0.10 | 5.37 ± 0.58 | 0.02 ± 0.06 | -0.75 ± 0.40 | 4.02 ± 0.69 | -1.33 ± 0.39 | -1.35 ± 0.39 | 5.35 ± 0.58 |
|  |  | 95% *CI* | 0.361 to 0.836 | 3.772 to 6.958 | -0.111 to 0.151 | -1.813 to 0.318 | 2.420 to 5.619 | -2.401 to -0.251 | -2.422 to -0.270 | 3.753 to 6.938 |
|  |  | *P*-value | < 0.001 | < 0.001 | 0.736 | 0.128 | < 0.001 | 0.027 | 0.025 | < 0.001 |
|  | *N. of* BC-BSIs *Reported to GLASS per Million Population* | MD ± SEM | 31.00 ± 5.13 | 119.80 ± 12.12 | 20.22 ± 4.59 | -69.35 ± 33.23 | 19.48 ± 35.00 | -80.13 ± 33.15 | -100.30 ± 32.87 | 99.61 ± 12.87 |
|  |  | 95% *CI* | 17.210 to 44.790 | 86.370 to 153.300 | 7.990 to 32.450 | -160.00 to 21.310 | -61.22 to 100.200 | -170.90 to 10.630 | -191.500 to -9.176 | 69.930 to 129.300 |
|  |  | *P*-value | 0.003 | < 0.001 | 0.009 | 0.102 | 0.593 | 0.071 | 0.038 | < 0.001 |
| **Burden of Clinically Significant Bacteria Causing**  **BC-BSIs** | Prevalence of Acinetobacter spp. from total BC-BSIs (%) | MD ± SEM | -11.86 ± 5.67 | -14.42 ± 5.58 | -10.10 ± 5.74 | -1.52 ± 3.03 | -4.09 ± 2.86 | 0.24 ± 3.16 | 10.34 ± 6.22 | -4.33 ± 1.53 |
|  |  | 95% *CI* | -27.130 to 3.399 | -29.800 to 0.949 | -25.300 to 5.110 | -8.521 to 5.473 | -11.830 to 3.659 | -7.045 to 7.533 | -4.010 to 24.690 | -7.866 to -0.792 |
|  |  | *P*-value | 0.099 | 0.060 | 0.145 | 0.629 | 0.222 | 0.940 | 0.135 | 0.022 |
|  | Prevalence of  S. aureus from total BC-BSIs (%) | MD ± SEM | -10.68 ± 7.08 | -6.64 ± 7.07 | -13.55 ± 7.23 | -12.43 ± 11.13 | -8.390 ± 11.12 | -15.30 ± 11.23 | -1.75 ± 13.12 | 6.912 ± 1.997 |
|  |  | 95% *CI* | -30.030 to 8.677 | -26.010 to 12.730 | -32.760 to 5.651 | -43.140 to 18.290 | -39.110 to 22.330 | -45.890 to 15.208 | -31.990 to 28.490 | 2.306 to 11.520 |
|  |  | *P-*value | 0.203 | 0.399 | 0.126 | 0.326 | 0.492 | 0.241 | 0.897 | 0.009 |
| **Resistant Priority Organisms Causing**  **BC-BSIs** | Prevalence of  BC-BSIs caused by CRAsp from BC-BSIs  caused by *Acinetobacter* *spp.* (%) | MD ± SEM | 4.06 ± 6.41 | 4.03 ± 6.07 | 4.71 ± 5.98 | -11.80 ± 3.77 | -11.82 ± 4.13 | -11.15 ± 4.02 | -15.86 ± 6.64 | -0.67 ± 3.24 |
|  |  | 95% *CI* | -14.930 to 23.050 | -10.320 to 18.390 | -9.426 to 18.830 | -20.490 to -3.112 | -21.340 to -2.308 | -20.420 to -1.888 | -31.570 to -0.149 | -8.132 to 6.792 |
|  |  | *P*-value | 0.566 | 0.528 | 0.457 | 0.014 | 0.021 | 0.024 | 0.048 | 0.841 |
|  | Prevalence of  BC-BSIs caused by CREC from BC-BSIs  caused by *E. coli* (%) | MD ± SEM | -2.04 ± 3.92 | -8.59 ± 3.67 | 5.05 ± 4.15 | 5.23 ± 1.53 | -1.38 ± 0.65 | 12.32 ± 2.05 | 7.27 ± 3.72 | -13.64 ± 1.95 |
|  |  | 95% *CI* | -11.070 to 7.005 | -18.76 to 1.576 | -4.520 to 14.62 | 1.694 to 8.770 | -3.088 to 0.4348 | 7.587 to 17.050 | -2.829 to 17.360 | -19.050 to -8.237 |
|  |  | *P*-value | 0.618 | 0.079 | 0.258 | 0.009 | 0.106 | < 0.001 | 0.118 | 0.002 |
|  | Prevalence of  BC-BSIs caused by 3GCREC from BC-BSIs caused by *E. coli* (%) | MD ± SEM | -19.36 ± 8.50 | -27.92 ± 8.50 | -4.77 ± 8.30 | 8.99 ± 1.67 | 0.43 ± 1.64 | 23.59 ± 3.50 | 28.35 ± 8.45 | -4.77 ± 8.30 |
|  |  | 95% *CI* | -45.670 to 6.939 | -54.240 to -1.600 | -24.400 to 14.870 | 5.141 to 12.840 | -3.350 to 4.212 | 14.360 to 32.810 | 1.850 to 54.860 | -24.400 to 14.870 |
|  |  | *P*-value | 0.103 | 0.043 | 0.584 | < 0.001 | 0.799 | 0.002 | 0.042 | 0.584 |
|  | Prevalence of  BC-BSIs caused by CRKP from BC-BSIs  caused by *K. pneumoniae* (%) | MD ± SEM | -9.12 ± 16.63 | -16.39 ± 16.64 | 0.52 ± 16.52 | 21.57 ± 4.43 | 14.30 ± 4.47 | 31.20 ± 3.97 | 30.69 ± 16.61 | -16.90 ± 4.11 |
|  |  | 95% *CI* | -75.990 to 57.750 | -83.140 to 50.370 | -67.530 to 68.560 | 11.350 to 31.790 | 3.986 to 24.620 | 22.050 to 40.360 | -36.400 to 97.780 | -26.380 to -7.423 |
|  |  | *P*-value | 0.635 | 0.422 | 0.978 | 0.001 | 0.013 | < 0.001 | 0.197 | 0.003 |
|  | Prevalence of  BC-BSIs caused by 3GCRKP from BC-BSIs caused by *K. pneumoniae* (%) | MD ± SEM | -15.79 ± 4.17 | -25.50 ± 4.74 | -1.22 ± 2.22 | -0.32 ± 4.81 | -10.04 ± 5.10 | 14.25 ± 4.01 | 15.47 ± 5.62 | -24.29 ± 3.31 |
|  |  | 95% *CI* | -25.990 to -5.588 | -37.110 to -13.900 | -6.642 to 4.208 | -11.410 to 10.760 | -21.790 to 1.720 | 3.390 to 25.110 | 1.709 to 29.230 | -33.150 to -15.420 |
|  |  | *P*-value | 0.009 | 0.002 | 0.603 | 0.948 | 0.085 | 0.021 | 0.033 | 0.001 |
|  | Prevalence of  BC-BSIs caused by MRSA from BC-BSIs  caused by *S. aureus* (%) | MD ± SEM | -31.07 ± 10.16 | -31.46 ± 10.04 | -30.08 ± 8.27 | -7.90 ± 15.19 | -8.29 ± 15.11 | -6.91 ± 15.32 | 23.17 ± 21.28 | -1.38 ± 3.48 |
|  |  | 95% *CI* | -70.880 to 8.746 | -72.350 to 9.433 | -50.320 to -9.838 | -49.340 to 33.540 | -49.830 to 33.250 | -48.210 to 34.390 | -28.910 to 75.250 | -9.411 to 6.653 |
|  |  | *P*-value | 0.081 | 0.082 | 0.011 | 0.629 | 0.612 | 0.674 | 0.318 | 0.703 |

**Abbreviations**: AMR = Antimicrobial Resistance, BC-BSIs = Bacteriologically Confirmed Bloodstream Infections, *CI* = Confidence Interval, CRAsp = Carbapenem-Resistant *Acinetobacter* spp., CREC = Carbapenem-Resistant *E. coli*, CRKP = Carbapenem-Resistant *K. pneumoniae*, MD = Mean of Differences, MRSA = Methicillin-Resistant *S. aureus*, SEM= Standard Error of Mean, 3GCREC = Third-Generation Cephalosporin-Resistant *E. coli*, 3GCRKP = Third-Generation Cephalosporin-Resistant *K. pneumoniae*.

**N.B.** *unpaired t-tests were used for group comparisons.

**Table S3.** Sensitivity Analysis: Antimicrobial Resistance (AMR) Surveillance Activities, Burden of Clinically Significant Bacteria Causing Bacteriologically Confirmed (BC) Bloodstream Infections (BSIs) (BC-BSIs), and Resistant Priority Organisms Causing BC-BSIs in WHO Eastern Mediterranean Region Countries, Classified by Conflict and Fragility Status **(Reported to GLASS, 2019–2021)**

| **Outcome** | | | **Category of Countries** | | | | |
| --- | --- | --- | --- | --- | --- | --- | --- |
|  |  |  | **Conflict  (*N* = 7  countries)** | **Fragile (*N* = 2  countries)** | **Non-conflict  (*N* = 13 countries)** | **Non-conflict  high income *(N* = 6 countries)** | **Non-conflict  low/middle income  (*N* = 7 countries)** |
| **AMR Surveillance Activities** | *N. of Sentinel Surveillance Sites per Million Population* | Mean | 0.20 | 2.13 | 0.91 | 6.36 | 0.25 |
|  |  | SD | 0.02 | 0.11 | 0.01 | 0.18 | 0.01 |
|  | *N. of* BC-BSIs *Reported to GLASS per Million Population* | Mean | 3.78 | 155.60 | 39.61 | 140.90 | 27.37 |
|  |  | SD | 2.62 | 20.96 | 5.79 | 8.49 | 6.35 |
| **Burden of Clinically Significant Bacteria Causing BC-BSIs** | Prevalence of Acinetobacter spp. from total BC-BSIs (%) | Mean | 26.07 | 11.97 | 13.21 | 10.16 | 15.06 |
|  |  | SD | 6.58 | 8.13 | 2.94 | 1.21 | 4.03 |
|  | Prevalence of S. aureus from total BC-BSIs (%) | Mean | 27.67 | 51.08 | 21.64 | 25.42 | 19.38 |
|  |  | SD | 1.67 | 2.79 | 2.21 | 2.48 | 4.63 |
| **Resistant Priority Organisms Causing BC-BSIs** | Prevalence of BC-BSIs  caused by CRAsp  from BC-BSIs  caused by *Acinetobacter* *spp.* (%) | Mean | 61.45 | 80.44 | 74.13 | 74.24 | 74.15 |
|  |  | SD | 3.03 | 6.58 | 0.92 | 4.68 | 3.53 |
|  | Prevalence of BC-BSIs  caused by CREC  from BC-BSIs  caused by *E. coli* (%) | Mean | 15.94 | 3.03 | 9.85 | 2.32 | 18.45 |
|  |  | SD | 5.26 | 1.49 | 3.83 | 0.32 | 3.72 |
|  | Prevalence of BC-BSIs  caused by 3GCREC  from BC-BSIs  caused by *E. coli* (%) | Mean | 79.57 | 47.76 | 58.95 | 49.78 | 72.95 |
|  |  | SD | 18.98 | 2.02 | 1.94 | 2.16 | 2.84 |
|  | Prevalence of BC-BSIs  caused by CRKP  from BC-BSIs  caused by *K. pneumoniae* (%) | Mean | 59.71 | 15.87 | 39.96 | 33.64 | 49.24 |
|  |  | SD | 18.24 | 8.06 | 3.10 | 3.82 | 1.48 |
|  | Prevalence of BC-BSIs  caused by 3GCRKP  from BC-BSIs  caused by *K. pneumoniae* (%) | Mean | 80.42 | 69.28 | 67.76 | 59.52 | 79.15 |
|  |  | SD | 4.72 | 6.53 | 3.92 | 4.57 | 1.06 |
|  | Prevalence of BC-BSIs  caused by MRSA  from BC-BSIs  caused by *S. aureus* (%) | Mean | 79.03 | 70.98 | 42.35 | 41.27 | 43.95 |
|  |  | SD | 10.37 | 8.80 | 2.18 | 1.81 | 3.13 |

**Abbreviations**: AMR = Antimicrobial Resistance, BC-BSIs = Bacteriologically Confirmed Bloodstream Infections, CRAsp = Carbapenem-Resistant *Acinetobacter* spp., CREC = Carbapenem-Resistant *E. coli*, CRKP = Carbapenem-Resistant *K. pneumoniae*, MRSA = Methicillin-Resistant *S. aureus*, N = Number, SD = Standard Deviation, 3GCREC = Third-Generation Cephalosporin-Resistant *E. coli*, 3GCRKP = Third-Generation Cephalosporin-Resistant *K. pneumoniae*.

**N.B.**

- For each outcome and category of countries, mean values and SD were calculated.

**Table S4.** Sensitivity Analysis: Group Comparisons of Countries Classified by Conflict and Fragility Status Across Study Outcomes Related to Antimicrobial Resistance Surveillance Activities, Burden of Clinically Significant Bacteria Causing Bacteriologically Confirmed Bloodstream Infections, and Resistant Priority Organisms in WHO Eastern Mediterranean Region Countries **(Reported to GLASS, 2019–2021)**

| **Outcome** | | | **Group Comparisons between categories of countries (Group A vs. B) (A-B)*** | | | | | | | |
| --- | --- | --- | --- | --- | --- | --- | --- | --- | --- | --- |
|  |  |  | **Non-conflict**  **vs.**  **Conflict** | **Non-conflict  high income**  **vs.**  **Conflict** | **Non-conflict  low/**  **middle income**  **vs.**  **Conflict** | **Non-conflict**  **vs.**  **Fragile** | **Non-conflict  high income**  **vs.**  **Fragile** | **Non-conflict  low/**  **middle income**  **vs.**  **Fragile** | **Conflict**  **vs.**  **Fragile** | **Non-conflict  high income**  **vs.**  **Non-conflict  low/**  **middle income** |
| **AMR Surveillance Activities** | *N. of Sentinel Surveillance Sites per Million Population* | MD ± SEM | 0.71 ± 0.01 | 6.16 ± 0.11 | 0.05 ± 0.01 | -1.22 ± 0.06 | 4.23 ± 0.12 | -1.89 ± 0.06 | -1.93 ± 0.06 | 6.11 ± 0.11 |
|  |  | 95% *CI* | 0.680 to 0.734 | 5.710 to 6.604 | 0.015 to 0.082 | -1.489 to -0.956 | 3.889 to 4.567 | -2.143 to -1.620 | -2.189 to -1.670 | 5.661 to 6.557 |
|  |  | *P*-value | <0.001 | <0.001 | 0.016 | 0.003 | <0.001 | <0.001 | <0.001 | <0.001 |
|  | *N. of* BC-BSIs *Reported to GLASS per Million Population* | MD ± SEM | 35.82 ± 3.67 | 137.20 ± 5.13 | 23.58 ± 3.96 | -116.00 ± 12.55 | -14.69 ± 13.05 | -128.30 ± 12.64 | -151.80 ± 12.19 | 113.60 ± 6.12 |
|  |  | 95% *CI* | 23.630 to 48.020 | 118.100 to 156.200 | 10.010 to 37.160 | -163.800 to  -68.280 | -50.930 to 21.560 | -175.400 to  -81.150 | -202.800 to  -100.900 | 96.590 to 130.600 |
|  |  | *P*-value | 0.003 | <0.001 | 0.013 | 0.007 | 0.324 | 0.005 | 0.006 | <0.001 |
| **Burden of Clinically Significant Bacteria Causing**  **BC-BSIs** | Prevalence of Acinetobacter spp. from total BC-BSIs (%) | MD ± SEM | -12.86 ± 4.16 | -15.91 ± 3.86 | -11.01 ± 4.46 | 1.25 ± 4.99 | -1.80 ± 4.75 | 3.10 ± 5.24 | 14.11 ± 6.04 | -4.90 ± 2.43 |
|  |  | 95% *CI* | -24.410 to -1.302 | -26.630 to -5.185 | -23.380 to 1.366 | -12.610 to 15.110 | -21.420 to 17.810 | -11.450 to 17.650 | -2.661 to 30.870 | -11.650 to 1.846 |
|  |  | *P*-value | 0.037 | 0.015 | 0.069 | 0.815 | 0.739 | 0.586 | 0.080 | 0.114 |
|  | Prevalence of  S. aureus from total BC-BSIs (%) | MD ± SEM | -6.02 ± 1.60 | -2.24 ± 1.73 | -8.29 ± 2.84 | -29.43 ± 2.06 | -25.65 ± 2.15 | -31.70 ± 3.12 | -23.41 ± 1.88 | 6.04 ± 3.03 |
|  |  | 95% *CI* | -10.470 to  -1.573 | -7.032 to 2.546 | -16.180 to -0.395 | -35.140 to  -23.730 | -31.630 to  -19.680 | -40.360 to  -23.030 | -28.620 to -18.200 | -2.373 to 14.460 |
|  |  | *P*-value | 0.020 | 0.263 | 0.043 | < 0.001 | < 0.001 | < 0.001 | < 0.001 | 0.117 |
| **Resistant Priority Organisms Causing**  **BC-BSIs** | Prevalence of  BC-BSIs caused by CRAsp from BC-BSIs  caused by *Acinetobacter* *spp.* (%) | MD ± SEM | 12.67 ± 1.82 | 12.79 ± 3.22 | 12.69 ± 2.68 | -6.31 ± 3.83 | -6.19 ± 4.66 | -6.29 ± 4.31 | -18.98 ± 4.18 | 0.10 ± 3.39 |
|  |  | 95% *CI* | 7.608 to 17.740 | 3.858 to 21.720 | 5.240 to 20.150 | -16.950 to 4.331 | -19.130 to 6.743 | -18.250 to 5.674 | -30.580 to -7.382 | -9.301 to 9.494 |
|  |  | *P*-value | 0.002 | 0.017 | 0.009 | 0.175 | 0.255 | 0.218 | 0.011 | 0.977 |
|  | Prevalence of  BC-BSIs caused by CREC from BC-BSIs  caused by *E. coli* (%) | MD ± SEM | -6.10 ± 3.76 | -13.63 ± 3.04 | 2.50 ± 3.72 | 6.82 ± 2.38 | -0.71 ± 0.88 | 15.42 ± 2.31 | 12.92 ± 3.16 | -16.13 ± 2.15 |
|  |  | 95% *CI* | -16.530 to 4.341 | -26.630 to -0.628 | -7.824 to 12.820 | 0.225 to 13.420 | -3.161 to 1.734 | 8.998 to 21.840 | 4.150 to 21.680 | -25.270 to -6.995 |
|  |  | *P*-value | 0.180 | 0.046 | 0.538 | 0.045 | 0.464 | 0.003 | 0.015 | 0.017 |
|  | Prevalence of  BC-BSIs caused by 3GCREC from BC-BSIs caused by *E. coli* (%) | MD ± SEM | -20.62 ± 11.01 | -29.79 ± 11.03 | -6.62 ± 11.08 | 11.19 ± 1.62 | 2.02 ± 1.71 | 25.19 ± 2.01 | 31.81 ± 11.02 | -23.17 ± 2.06 |
|  |  | 95% *CI* | -67.090 to 25.850 | -76.110 to 16.540 | -52.390 to 39.150 | 6.698 to 15.690 | -2.714 to 6.762 | 19.610 to 30.780 | -14.610 to 78.230 | -28.880 to  -17.450 |
|  |  | *P*-value | 0.200 | 0.111 | 0.609 | 0.002 | 0.301 | <0.001 | 0.099 | <0.001 |
|  | Prevalence of  BC-BSIs caused by CRKP from BC-BSIs  caused by *K. pneumoniae* (%) | MD ± SEM | -19.74 ± 9.89 | -26.07 ± 10.03 | -10.46 ± 9.68 | 24.09 ± 4.98 | 17.77 ± 5.15 | 33.37 ± 4.73 | 43.83 ± 11.33 | -15.60 ± 2.37 |
|  |  | 95% *CI* | -51.200 to 11.720 | -57.970 to 5.842 | -41.260 to 20.330 | 10.260 to 37.920 | 3.474 to 32.060 | 20.240 to 46.500 | 7.763 to 79.900 | -22.170 to -9.028 |
|  |  | *P*-value | 0.140 | 0.080 | 0.359 | 0.008 | 0.026 | 0.002 | 0.031 | 0.003 |
|  | Prevalence of  BC-BSIs caused by 3GCRKP from BC-BSIs caused by *K. pneumoniae* (%) | MD ± SEM | -12.66 ± 3.54 | -20.90 ± 3.78 | -1.27 ± 2.79 | -1.52 ± 4.40 | -9.75 ± 4.59 | 9.87 ± 3.82 | 11.14 ± 4.65 | -19.62 ± 2.68 |
|  |  | 95% *CI* | -22.490 to -2.823 | -31.370 to  -10.420 | -9.021 to 6.477 | -13.730 to 10.700 | -22.490 to 2.984 | -0.736 to 20.480 | -1.773 to 24.060 | -27.070 to  -12.170 |
|  |  | *P*-value | 0.023 | 0.005 | 0.672 | 0.748 | 0.101 | 0.061 | 0.075 | 0.002 |
|  | Prevalence of  BC-BSIs caused by MRSA from BC-BSIs  caused by *S. aureus* (%) | MD ± SEM | -36.68 ± 5.70 | -37.76 ± 5.63 | -35.08 ± 5.94 | -28.63 ± 5.24 | -29.71 ± 5.19 | -27.03 ± 5.39 | 8.05 ± 8.54 | -2.68 ± 2.08 |
|  |  | 95% *CI* | -54.830 to  -18.540 | -55.680 to  -19.850 | -53.990 to -16.170 | -43.170 to  -14.100 | -44.120 to  -15.310 | -42.000 to  -12.060 | -19.130 to 35.230 | -8.470 to 3.105 |
|  |  | *P*-value | 0.008 | 0.007 | 0.010 | 0.005 | 0.005 | 0.007 | 0.415 | 0.268 |

**Abbreviations**: AMR = Antimicrobial Resistance, BC-BSIs = Bacteriologically Confirmed Bloodstream Infections, CI = Confidence Interval, CRAsp = Carbapenem-Resistant *Acinetobacter* spp., CREC = Carbapenem-Resistant *E. coli*, CRKP = Carbapenem-Resistant *K. pneumoniae*, MD = Mean of Differences, MRSA = Methicillin-Resistant *S. aureus*, SEM= Standard Error of Mean, 3GCREC = Third-Generation Cephalosporin-Resistant *E. coli*, 3GCRKP = Third-Generation Cephalosporin-Resistant *K. pneumoniae*.

**N.B.** *unpaired t-tests were used for group comparisons.

**Table S5.** Sensitivity Analysis: Antimicrobial Resistance (AMR) Surveillance Activities and Burden of Clinically Significant Bacteria Causing Bacteriologically Confirmed (BC) Bloodstream Infections (BSIs) (BC-BSIs) in WHO Eastern Mediterranean Region Countries, Classified by Conflict and Fragility Status (Reported to GLASS, 2017–2021) - **Random Country Selection Approach**

| **Outcome** | | | **Category of Countries** | | | | |
| --- | --- | --- | --- | --- | --- | --- | --- |
|  |  |  | **Conflict  (*N* = 7  countries)** | **Fragile (*N* = 2  countries)** | **Non-conflict  (*N* = 13 countries)** | **Non-conflict  high income (*N* = 6 countries)** | **Non-conflict  low/middle income  (*N* = 7 countries)** |
| **AMR Surveillance Activities** | *N. of Sentinel Surveillance Sites per Million Population* | Mean | 0.20 | 1.51 | 1.03 | 5.24 | 0.20 |
|  |  | SD | 0.10 | 0.87 | 0.25 | 1.07 | 0.10 |
|  | *N. of* BC-BSIs *Reported to GLASS per Million Population* | Mean | 4.09 | 103.20 | 48.86 | 117.70 | 35.11 |
|  |  | SD | 4.00 | 73.45 | 14.66 | 15.53 | 15.05 |
| **Burden of Clinically Significant Bacteria Causing BC-BSIs** | Prevalence of Acinetobacter spp. from total BC-BSIs (%) | Mean | 24.53 | 13.72 | 10.78 | 9.77 | 11.57 |
|  |  | SD | 12.66 | 6.29 | 2.88 | 1.29 | 3.97 |
|  | Prevalence of S. aureus from total BC-BSIs (%) | Mean | 30.87 | 33.04 | 20.27 | 25.25 | 16.66 |
|  |  | SD | 15.85 | 24.79 | 2.85 | 2.85 | 4.90 |

**Abbreviations**: AMR = Antimicrobial Resistance, BC-BSIs = Bacteriologically Confirmed Bloodstream Infections, N = Number, SD = Standard Deviation.

**N.B.**

- For each outcome and category of countries, mean values and SD were calculated.

**Table S6.** Sensitivity Analysis: Group Comparisons of Countries Classified by Conflict and Fragility Status Across Study Outcomes Related to Antimicrobial Resistance Surveillance Activities and Burden of Clinically Significant Bacteria Causing Bacteriologically Confirmed Bloodstream Infections in WHO Eastern Mediterranean Region Countries (Reported to GLASS, 2017–2021) - Random Country Selection Approach

| **Outcome** | | | **Group Comparisons between categories of countries (Group A vs. B) (A-B)*** | | | | | | | |
| --- | --- | --- | --- | --- | --- | --- | --- | --- | --- | --- |
|  |  |  | **Non-conflict**  **vs.**  **Conflict** | **Non-conflict  high income**  **vs.**  **Conflict** | **Non-conflict  low/**  **middle income**  **vs.**  **Conflict** | **Non-conflict**  **vs.**  **Fragile** | **Non-conflict  high income**  **vs.**  **Fragile** | **Non-conflict  low/**  **middle income**  **vs.**  **Fragile** | **Conflict**  **vs.**  **Fragile** | **Non-conflict  high income**  **vs.**  **Non-conflict  low/**  **middle income** |
| **AMR Surveillance Activities** | *N. of Sentinel Surveillance Sites per Million Population* | MD ± SEM | 0.84 ± 0.12 | 5.04 ± 0.48 | -0.003 ± 0.06 | -0.47 ± 0.40 | 3.73 ± 0.62 | -1.31 ± 0.39 | -1.31 ± 0.39 | 5.04 ± 0.48 |
|  |  | 95% *CI* | 0.564 to 1.108 | 3.716 to 6.361 | -0.147 to 0.141 | -1.535 to 0.591 | 2.311 to 5.150 | -2.386 to -0.236 | -2.383 to -0.233 | 3.720 to 6.364 |
|  |  | *P*-value | < 0.001 | < 0.001 | 0.960 | 0.299 | < 0.001 | 0.027 | 0.028 | < 0.001 |
|  | *N. of* BC-BSIs *Reported to GLASS per Million Population* | MD ± SEM | 44.77 ± 6.79 | 113.60 ± 7.17 | 31.01 ± 6.96 | -54.35 ± 33.49 | 14.47 ± 33.57 | -68.11 ± 33.53 | -99.12 ± 32.90 | 82.58 ± 9.67 |
|  |  | 95% *CI* | 26.820 to 62.710 | 94.560 to 132.600 | 12.590 to 49.440 | -144.700 to 36.000 | -75.810 to 104.800 | -158.400 to 22.220 | -190.200 to -7.997 | 60.280 to 104.900 |
|  |  | *P*-value | 0.002 | < 0.001 | 0.008 | 0.175 | 0.687 | 0.107 | 0.039 | <0.001 |
| **Burden of Clinically Significant Bacteria Causing**  **BC-BSIs** | Prevalence of Acinetobacter spp. from total BC-BSIs (%) | MD ± SEM | -13.74 ± 5.81 | -14.76 ± 5.69 | -12.96 ± 5.94 | -2.94 ± 3.09 | -3.95 ± 2.87 | -2.15 ± 3.33 | 10.81 ± 6.32 | -1.80 ± 1.87 |
|  |  | 95% *CI* | -29.290 to 1.804 | -30.440 to 0.919 | -28.430 to 2.514 | -10.070 to 4.195 | -11.690 to 3.780 | -9.819 to 5.519 | -3.774 to 25.390 | -6.108 to 2.502 |
|  |  | *P*-value | 0.071 | 0.059 | 0.083 | 0.370 | 0.235 | 0.536 | 0.126 | 0.363 |
|  | Prevalence of  S. aureus from total BC-BSIs (%) | MD ± SEM | -10.60 ± 7.20 | -5.62 ± 7.20 | -14.21 ± 7.42 | -12.77 ± 11.16 | -7.79 ± 11.16 | -16.39 ± 11.30 | -2.18 ± 13.16 | 8.60 ± 2.54 |
|  |  | 95% *CI* | -30.120 to 8.933 | -25.140 to 13.910 | -33.580 to 5.159 | -43.450 to 17.900 | -38.470 to 22.880 | -46.890 to 14.110 | -32.520 to 28.170 | 2.7460 to 14.440 |
|  |  | *P*-value | 0.211 | 0.477 | 0.117 | 0.315 | 0.523 | 0.216 | 0.873 | 0.001 |

**Abbreviations**: AMR = Antimicrobial Resistance, BC-BSIs = Bacteriologically Confirmed Bloodstream Infections, CI = Confidence Interval, MD = Mean of Differences, SEM= Standard Error of Mean.

**N.B.** *unpaired t-tests were used for group comparisons.
